# The Rapid Sense of Direction (R-SOD) Scale: A Brief Self-Report Tool to Identify Developmental Topographical Disorientation (DTD)

**DOI:** 10.3390/brainsci15060622

**Published:** 2025-06-09

**Authors:** Tejdeep Jaswal, Ford Burles, Giuseppe Iaria

**Affiliations:** NeuroLab, Canadian Space Health Research Network, Department of Psychology, Hotchkiss Brain Institute, Alberta Children’s Hospital Research Institute, University of Calgary, Calgary, AB T2N 1N4, Canada; tejdeep.jaswal@ucalgary.ca (T.J.); cfburles@ucalgary.ca (F.B.)

**Keywords:** cognitive map, getting lost, memory, questionnaire, space, wayfinding

## Abstract

**Background/Objectives:** Developmental Topographical Disorientation (DTD) refers to a condition in which individuals report getting lost in very familiar surroundings, since childhood, with no other cognitive complaints, and no brain injuries or neurological disorders. While the cognitive and neurological mechanisms underlying DTD are being investigated, to date, there is no tool available to the public and health practitioners for identifying this lifelong condition. **Methods**: Here, we used a decade of data (*N* = 3794) collected in healthy and DTD individuals to produce a short and reliable measure of self-reported sense of direction that could point to the presence of DTD. **Results**: We adopted a measure of internal consistency (Cronbach’s alpha) and identified four items of the well-known Santa Barbara Sense of Direction (SBSOD) Scale that retain its original strong internal consistency. These four items remain sensitive to the well-known effects of sex on spatial orientation and, importantly, to the presence of DTD, while maintaining the same pattern of association with a cognitive battery of computerized tasks measuring different spatial abilities. **Conclusions**: This four-item measure could be of practical use to obtain a rapid assessment of an individual’s self-reported sense of direction and help to identify the presence of DTD in the general population.

## 1. Introduction

Spatial orientation is an essential skill for daily life since it allows individuals to navigate efficiently within the environment in an independent manner and without getting lost [1]. Spatial orientation relies on the sense of direction, which is defined as the individuals’ awareness of their location within the environment and orientation towards unseen landmarks within it [2]. Generally, people are aware of their sense of direction and are able to provide an accurate estimate of it [3].

Several large-scale spatial skills contribute to one’s sense of direction. They include the ability to read maps [2], give and follow directions [4], learning routes [3], estimate distances [2], point to unseen landmarks [2,3], and navigate efficiently [5]. Moreover, one’s sense of direction involves landmarks’ familiarity [3], with orientation to landmarks likely playing a critical role in maintaining sense of direction [6]. Altogether, these skills provide individuals with the complex trait of sense of direction that defines one’s ability to orient and navigate in spatial surroundings.

The trait of sense of direction has primarily been examined through self-report assessments. One widely used and well-known self-report measure of an individual’s sense of direction is the Santa Barbara Sense of Direction (SBSOD) Scale [4]. This scale has high internal reliability, with a Cronbach’s alpha of 0.88 reported in the original study [4] and a value of 0.87 reported more recently [7]. Furthermore, the scale shows good item–total correlations ranging from 0.34 to 0.75, as well as an overall high test–retest reliability of 0.90, with individual test–retest reliability correlations ranging from 0.42 to 0.90 [4], suggesting that the scale demonstrates strong reliability. The SBSOD scale has also been shown to correlate with several tasks, including those measuring the ability to point to environmental landmarks, update one’s position in space while blindfolded, and estimate directions and distances [4]. Additionally, the scale is related to the ability to update and assess one’s orientation and location in a new environment [4]; these relationships indicate the validity of the SBSOD scale, as they demonstrate its connection to several large-scale spatial abilities. Overall, the SBSOD scale is a validated and reliable measure that effectively quantifies the construct of sense of direction of the individuals.

Aside from the individual variability in orienting and navigating spatial surroundings within the healthy population [8], some individuals suffer from a lifelong condition that makes them lost even in extremely familiar surroundings, a condition known as Developmental Topographical Disorientation (DTD) [9]. In individuals affected by DTD, besides the selective difficulty in orienting, cognitive functions remain intact, and no brain damage or neurological conditions are reported [10,11,12,13]. All cases of DTD described so far in the literature report the same symptoms: getting lost in familiar surroundings, difficulties with orientation experienced since childhood, no cognitive or memory impairments, and no brain injuries or neurological disorders [14]. For most individuals, their difficulty with orienting is related to their inability to form a mental representation of their environment (i.e., a cognitive map; [12,14,15,16,17]). Case studies of DTD have also highlighted the impact of the condition on individual’s everyday life, their professional career, and their social relationships [10,11,17,18]. Despite the knowledge gained on the behavioral, cognitive, and neurological mechanisms underlying DTD, to date, there is no tool available to the public and health practitioners to identify in the general population such a devastating lifelong developmental condition.

Previous work [16] has demonstrated that individuals with DTD report a poorer sense of direction as measured by the SBSOD scale compared to healthy controls, indicating that this scale is able to discriminate between individuals with DTD and individuals without severe topographical disorientation. However, while the SBSOD scale is effective as a broad measure of sense of direction, it was not designed specifically for identifying DTD, nor optimized for brevity or clinical application. In the present study, we extend the utility of the SBSOD by systematically identifying a subset of items that retain the core psychometric strengths of the original scale while offering improved practicality for rapid screening. Specifically, we apply a rigorous item reduction process using internal consistency metrics and factor analysis to develop a concise 4-item tool—the Rapid Sense of Direction (R-SOD) scale—that preserves sensitivity to sex differences and group status (DTD vs. control) and maintains correlations with objective spatial tasks. This innovation provides a novel, efficient, and scalable self-report measure tailored for detecting DTD in both research and clinical settings. In assessing the nomological net (the outcomes the construct is related to, [19]) of such a subset of items, we further expect them (as a measure of sense of direction) to correlate with large-scale spatial functions such as the ability to form cognitive maps and the ability to recognize places from different perspectives (i.e., perspective-taking skill), which are known to be critical for successful navigation and orientation in large-scale spatial surroundings [20,21]. The R-SOD thus represents a refinement of the SBSOD with targeted application to DTD, transforming a general assessment tool into a focused diagnostic screening instrument.

## 2. Materials and Methods

### 2.1. Participants

We recruited and tested 3794 participants through our online platform (www.gettinglost.ca) from 1 October 2009 to 30 September 2023. This sample included participants that were recruited from an online Research Participation System (RPS) through the University of Calgary as well as individuals who completed the testing unsolicited online. Recruitment methods were consistent over the years. This sample included 2201 healthy individuals who did not self-report any problems with orientation, as well as no cognitive complaints, brain injuries, or neurological conditions; among this sample of healthy participants, 1419 were females (Mean age = 30.61 years, SD = 13.93 years) and 782 were males (Mean age = 28.46 years, SD = 12.23). In this group, 1102 participants (50.1%) completed the testing through RPS, and 1099 participants completed the testing unsolicited (49.9%). The large sample of 3794 participants also included 1593 individuals who self-reported to be affected by Developmental Topographical Disorientation (DTD); this group of DTD individuals consisted of 1312 females (Mean age = 35.85 years, SD = 13.21 years) and 281 males (Mean age = 35.10 years, SD = 12.25 years). For this group, 115 participants (7.2%) were recruited through RPS, and 1478 individuals (92.8%) completed the testing unsolicited. Consistent with the inclusion/exclusion criteria for DTD [11], individuals in the DTD group reported (a) getting lost often in very familiar surroundings, (b) experiencing problems with orientation from childhood or adolescence, (c) reporting no other cognitive complaints (attentional, perceptual, or memory issues), and (d) no brain injury/neurological conditions. Participants were more likely to be recruited through RPS in the healthy participants group compared to the DTD group (χ^2^ = 778.77, *p* < 0.001, Odds Ratio = 12.89). In other words, the DTD group was skewed towards recruitment through unsolicited testing. In the healthy participants group, females had lower odds of being recruited through RPS compared to males (χ^2^ = 18.64, *p* < 0.001, Odds Ratio = 0.68). In the DTD group, there was no association between recruitment method and sex (χ^2^ = 1.80, *p* = 0.179). Participants recruited through RPS were compensated with course credits. There was no compensation for participants recruited through unsolicited testing. This study was approved by the local research ethics board.

### 2.2. Procedure

All participants included in the study completed a demographic questionnaire and the Santa Barbara Sense of Direction (SBSOD) scale [4]. In addition, participants performed a series of computerized cognitive tasks, providing an objective evaluation of a variety of cognitive skills. Hereafter is a more detailed description of each measure.

#### 2.2.1. Santa Barbara Sense of Direction (SBSOD) Scale

The scale consists of 15 items that require a self-assessment of sense of direction, feelings towards traveling, concerns about getting lost, and exploring the environment [4]. For each item on this scale, participants report their level of agreement on a Likert scale ranging from 1 (strongly agree) to 7 (strongly disagree). Approximately half of the items are stated positively while the remaining items are stated negatively [4]. In this study, positively stated items have their scores reversed so that a higher score corresponds to a stronger self-reported sense of direction. All participants included in the study completed this scale.

#### 2.2.2. Cognitive Battery

**Figure 1:**
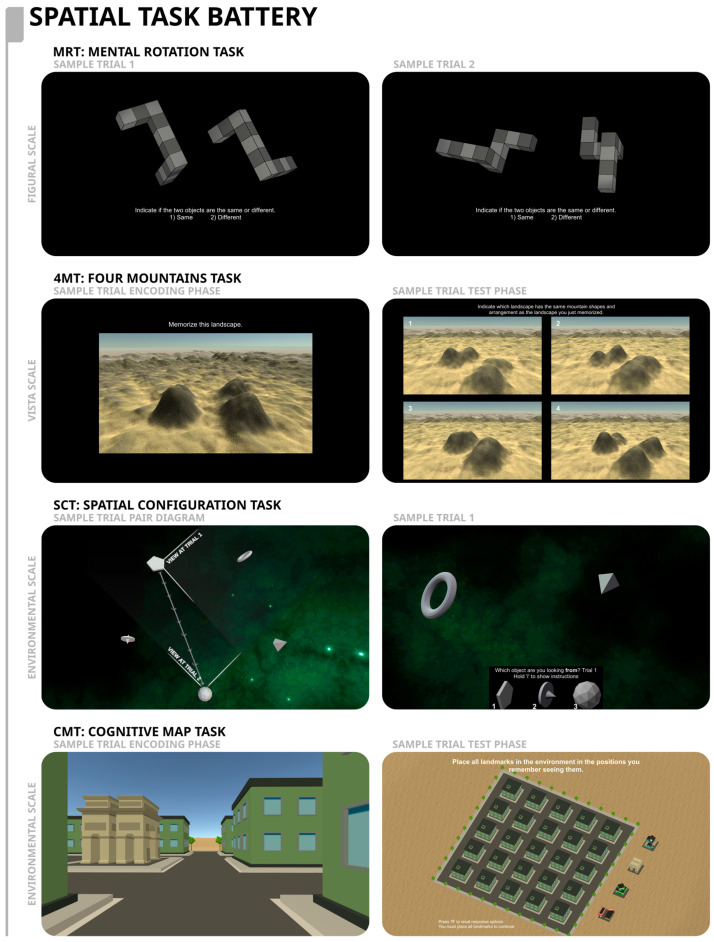
The top row displays the Mental Rotation Task (MRT), with the sample stimulus presented in the in-task instructions with sample trial 1 (**left panel**) and sample trial 2 (**right panel**). The second row displays the Four Mountains Task (4MT), with a view of the sample stimulus to be memorized (**left panel**), and a sample set of four response options, with the target response being the fourth option (**right panel**). The third row shows the Spatial Configuration Task with a view of the camera movement between two sample trials (**left panel**), and the user’s view from “Trial 1”, where Option 1 is the correct response (**right panel**). The overview displayed in the left panel is not shown to the participants. The bottom row displays the Cognitive Map Task (CMT), a view of one of the landmarks seen in the environment (**left panel**) and a response screen shown to the participant (**right panel**). These figures are derivative works of materials provided by Ford Burles under a CC BY 4.0 license.

The cognitive battery we used included a variety of different spatial tasks (Figure 1) as well as a face memory task. This battery has been used and described in our previous work [16], and includes participants enrolled in a variety of studies. As such, there are varying degrees of completeness across the spatial tasks. The spatial tasks capture performance across different spatial scales [22,23] and included tasks at the small or figural scale (i.e., the mental rotation task), an intermediate or meso scale (i.e., the four mountains task), and the large or environmental scale (i.e., the spatial configuration and cognitive map tasks). Reliability estimates for these tasks can be found in Table A1 in Appendix A.

##### Cambridge Face Memory Test

We asked participants to perform the Cambridge Face Memory Test (CFMT) [24], which assesses the ability of individuals to memorize and recognize faces. The task was completed by 1202 healthy (683 females; Mean age = 28.66 years, SD = 12.96 years, 519 males; Mean age = 26.87 years, SD = 11.72 years) and 596 DTD (487 females; Mean age = 35.67 years, SD = 13.07 years, 109 males; Mean age = 35.17 years, SD = 11.52 years) participants. The test requires participants to first memorize six different faces (one at a time) and then recognize each of these faces among three other faces that are presented simultaneously [24]. Performance is measured by scoring the number of correct responses. This task was included as a non-spatial control task, as we have no evidence this task should be directly associated with spatial ability. Associations between self-reported sense of direction and performance on this task are used to represent noise floor associations between subjective assessment and unrelated objective cognitive performance.

##### Mental Rotation Task

The Mental Rotation Task (MRT) was completed by 1350 healthy (805 females; Mean age = 26.37 years, SD = 11.90 years, 545 males; Mean age = 26.10 years, SD = 11.32 years) and 575 DTD (479 females; Mean age = 34.91 years, SD = 13.31 years, 96 males; Mean age = 35.66 years, SD = 11.74 years) participants. This task assesses the ability to mentally rotate objects [25], a skill that is critical for spatial orientation and navigation. The task consisted of 80 trials. In each trial, participants have to determine if two three-dimensional objects presented side by side are the same object or not, irrespective of the magnitude of rotation between the two objects. The two objects are showcased at a range of rotational disparities, and the participants must mentally rotate the objects into congruence and determine if they are the same or different [16]. Performance was measured as the proportion of correct responses.

##### Four Mountains Task

The Four Mountains Task (4MT) was completed by 1337 healthy (796 females; Mean age = 26.14 years, SD = 11.84 years, 541 males; Mean age = 25.69 years, SD = 11.21 years) and 547 DTD (457 females; Mean age = 35.11 years, SD = 13.46 years, 90 males; Mean age = 35.47 years, SD = 11.60 years) participants. The task examines the perspective-taking ability, that is, the ability to recognize a scene from a different viewpoint [16]. The task consists of 20 trials (see Figure 1). In each trial, participants are presented with a landscape showing four mountains for a duration of eight seconds and required to memorize that given landscape [16]; this is then followed by 2 s of a blank screen, after which four different landscapes are shown and participants are required to identify the landscape that they had memorized. One of these landscapes depicts the scene presented initially from a different perspective, and the other three options have an arrangement that is different from the image presented initially. To select their response, participants are given unlimited time. We measured the number of correct responses.

##### Spatial Configuration Task

This task was performed by 1280 healthy (783 females; Mean age = 25.58 years, SD = 11.33 years, 497 males; Mean age = 25.53 years, SD = 11.04 years) and 495 DTD (414 females; Mean age = 34.78 years, SD = 13.41 years, 81 males; Mean age = 33.90 years, SD = 11.39 years) participants. The Spatial Configuration Task (SCT) examines the ability to create a mental representation of the environment (i.e., a cognitive map) [15] by asking participants to form a mental representation of the spatial relationship between landmarks available in a virtual environment [26]. The task consists of 60 trials performed in a space-like virtual environment composed of five geometric objects placed pseudorandomly in the shape of a pentagon [16] (see Figure 1). The location of the objects remains constant throughout the task which the participants were informed about before beginning the task. In each trial, participants are shown a perspective of the camera on top of one of the objects while they are viewing two other objects [16]. Participants are required to use the keyboard to indicate which object the camera is looking from as they are presented with three response objects at the bottom of the screen [16]. After participants provide their answer, the camera moves to another object, and they are required again to indicate which objects the camera is looking from (with again three objects/options presented at the bottom of the screen). We measured accuracy over the 60 trials.

##### Cognitive Map Task (CMT)

The Cognitive Map Task (CMT) was performed by 620 healthy (344 females; Mean age = 19.92 years, SD = 4.53 years, 276 males; Mean age = 20.01 years, SD = 2.60 years) and 38 DTD (30 females; Mean age = 21.17 years, SD = 4.21 years, 8 males; Mean age = 22.75 years, SD = 6.96 years) participants. This test, as the one described above, also assesses the ability of individuals to generate and use a mental representation of the environment (and the spatial relationship between environmental landmarks) (i.e., a cognitive map) [15] for the purpose of orientation and navigation [27]. However, in this task, the environment consists of a virtual city composed of a 5 × 5 rectangular grid of buildings, with four distinct environmental landmarks [27] (see Figure 1). The task consists of a maximum number of 12 trials. In each trial, participants are presented with a video of a person moving in a virtual neighborhood, with brief pauses at each of the four distinct landmarks that they encounter; at the end of the video, participants are presented with a birds-eye view of the environment and asked to indicate the locations of the four landmarks. The task ends either when participants have indicated the correct locations of the four landmarks on the map or when the 12 trials elapsed. The virtual environment remains unchanged throughout the 12 trials. We measured the number of trials required to solve the task [16]; scores were transformed such that higher scores are indicative of better performance.

#### 2.2.3. SBSOD Scale Shortening Procedure

We used Cronbach’s alpha [28] as the primary feature to identify the ideal minimal number of items that is able to identify the presence of DTD and retains the same strong internal consistency and reliability of the full SBSOD scale. Beginning with the original 15 items, we consecutively dropped the item with the least positive contribution to the scale wise alpha. For the final set of items, we selected the semi-arbitrary ‘excellent internal consistency’ [29,30,31] threshold of α = 0.9. To supplement the removal of items using Cronbach’s alpha, we also conducted a confirmatory factor analysis to examine factor loadings across the items. Similarly to Cronbach’s alpha, we consecutively dropped the item with the lowest factor loading. To ensure the final set of items measure a single factor, we conducted an exploratory factor analysis with the final items. First, at the item level for descriptive purposes, and then at each step of item selection, we reported measures of association with sex and DTD status (Cohen’s D), as well as with performance on each task (Pearson’s r). Considering that sex effects are largely reported in the healthy population [32,33,34] and to mitigate the sex bias in DTD [16], sex effects are only reported in the control healthy group. For the same reason, the effect of DTD status is reported with the effect of sex removed. Finally, we also reported the association between scale/item and performance on our tasks of the cognitive battery using partial correlation coefficients controlling for both sex and DTD status. Partial correlation coefficients were included to demonstrate the unique variance of sense of direction beyond DTD status and sex to ensure that the resultant scales can be evaluated on these features independently, given the known associations between sex, topographical disorientation, and performance on these different tasks [16].

## 3. Results

### 3.1. Group Effects and Associations with Behavioral Performance

**Figure 2:**
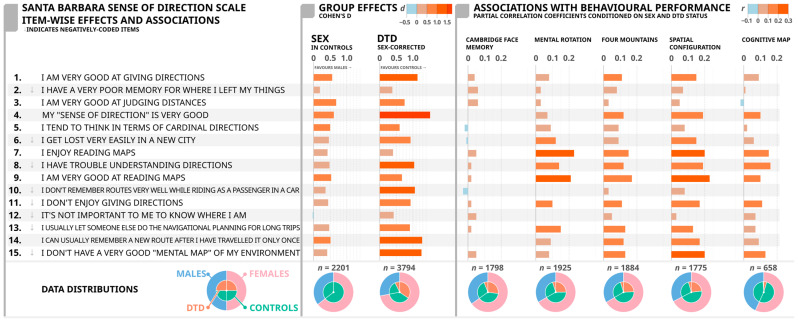
Cohen’s d (***d***) values for sex (females and males) and group (DTD and healthy controls) as well as partial correlations (***r***) (controlled for sex and DTD) of each SBSOD item (gray arrows indicate statements negatively worded), and the total scale score with Cambridge Face Memory Task (CFMT), Mental Rotation Task (MRT), Four Mountains Task (4MT), Spatial Configuration Task (SCT), and Cognitive Map Task (CMT). The colors are on a spectrum from blue to orange where blue represents negative values of Cohen’s d and partial correlation coefficients and orange represents positive values of Cohen’s d and partial correlation coefficients. The darker the color, the stronger the Cohen’s d and partial correlation coefficient value. For the data distributions, green represents healthy individuals, orange represents DTD individuals, blue represents males, and pink represents females.

First, we investigated the item-wise associations between the 15 different SBSOD scale statements and sex, DTD status, and task performance (see Figure 2). As expected, [35,36,37], we found that females generally reported worse evaluations of their own spatial abilities as compared to males; the only exception to this finding is Item 12 (“It’s not important to me to know where I am”), in which the sex difference was negligible. In contrast, Item 3 (“I am very good at judging distances”) showed the largest Sex effect size (d = 0.68) among all of the items, with males self-reporting better judgment of distances as compared to females.

With respect to the DTD effects, individuals with DTD reported a lower score on all items, confirming worse self-reported measures as compared to healthy individuals [16]. Most of the items depicted medium to large effect sizes (i.e., ds exceeding 0.50) with the exception of Item 2 (“I have a poor memory for where I left things”), Item 7 (“I enjoy reading maps), and Item 12 (“It’s not important to me to know where I am”), in which only small effects differentiated the two groups. On the other hand, and importantly, we found very large effect sizes on Item 1 (“I am very good at giving directions”), Item 4 (“My “sense of direction” is very good), Item 8 (“I have trouble understanding directions”), Item 10 (“I don’t remember routes very well while riding as a passenger in a car”), Item 14 (“I can usually remember a new route after I have traveled it only once”), and Item 15 (“I don’t have a very good “mental map” of my environment”).

As expected, the measures collected for the non-spatial face memory task (i.e., the CFMT) showed very small partial correlations with all of the items. On the other hand, we found that the SCT had the strongest correlation with most of the items. The 4MT also consistently showed stronger correlations with most of the items. The MRT seemed to display relatively smaller associations with almost all of the items except for the map-reading items (Item 7: “I enjoy reading maps”, and Item 9: “I am very good at reading maps”). The CMT depicted stronger correlations with a few items such as Item 7 (“I am very good at reading maps”) and Item 8 (“I have trouble understanding directions”). The highest correlation observed in our dataset was the SCT with Item 9 (“I am very good at reading maps”) and MRT with Item 7 (“I enjoy reading maps”). Items that showed weak correlations with most of the tasks included Item 2 (“I have a poor memory for where I left my things”), Item 3 (“I am very good at judging distances”), Item 5 (“I tend to think of my environment in terms of cardinal directions”), Item 10 (“I don’t remember routes very well while riding as a passenger in a car”), and Item 12 (“It’s not important to me to know where I am”). With the overall scale score, the strongest correlation was reported with the SCT, followed by the 4MT, MRT, and CMT. Correlations between the non-spatial task (i.e., the CFMT) and most of the items, as expected, were not effectively significant. This demonstrates evidence of the scale’s validity since the scale correlates with constructs related to sense of direction (4MT, SCT, and CMT) and does not correlate with unrelated constructs related to face processing (CFMT). These findings are displayed in Figure 2.

### 3.2. Scale Shortening

**Figure 3:**
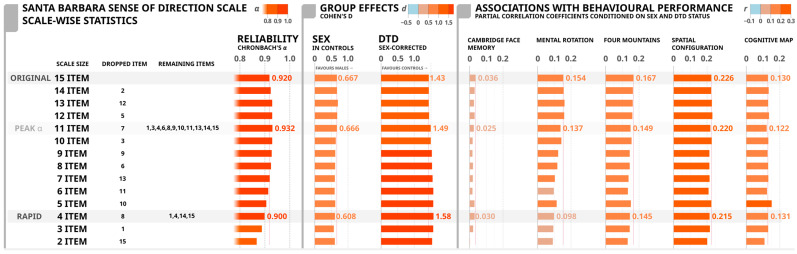
Properties of the shortened scales include Cohen’s D (***d***) values to indicate the differences due to sex and DTD status, partial correlation coefficients (***r***) with nonspatial (Cambridge Face Memory Task (CFMT)) and spatial orientation tasks (Mental Rotation Task (MRT), Four Mountains Task (4MT), Spatial Configuration Task (SCT), Cognitive Map Task (CMT)), and internal consistency (Cronbach’s alpha, α). Item dropped indicates the item dropped in order to create the shortened scale in the corresponding row. The colors are on a spectrum where lighter colors represent a lower Cronbach’s alpha, Cohen’s d, and partial coefficient correlation value whereas darker colors represent a higher Cronbach’s alpha, Cohen’s d, and partial coefficient correlation value.

Our stepwise scale shortening effects are reported in Figure 3. This shortening procedure generated improvements or stable internal consistencies up to a 11-item version (excludes items 2, 5, 7, and 12) of the scale, generating the most consistent item set with an alpha of 0.932. Continued shortening produced reductions in alpha scores, but these scales continued to display “excellent” internal consistency [29,30,31] through the 4-item version of the scale. This 4-item version of the scale includes original items 1 (“I am very good at giving directions”), 4 (“My sense of direction is very good”), 14 (“I can usually remember a new route after I have traveled it only once”), and 15 (“I don’t have a very good mental map of my environment”), and includes positively and negatively worded items. We will refer to this shortened scale as ‘Rapid Sense of Direction (R-SOD) Scale.

The R-SOD scale generally retains the same pattern of associations as the full scale. Differences between males and females remained strong (d from 0.667 to 0.608 in SBSOD and R-SOD, respectively), as did the differences between individuals identified as DTD and healthy controls (d from 1.435 to 1.576). The associations with the face memory task remained small (r from 0.036 to 0.030), and the associations with the 4MT, SCT, and CMT remained stable (rs from 0.167 to 0.145, 0.226 to 0.215, 0.130 to 0.131, respectively). The sole departure from common properties with the full scale was the reduction in association with performance in the mental rotation task (r from 0.154 to 0.098); this is largely due to the removal of the map-reading items (7 and 9), which showed the strongest associations with this task. Perhaps unsurprisingly, if this stepwise removal procedure continues to where it can no longer proceed, one of the two remaining items is the statement that shares the same terminology as the scale title, i.e., item 4, ‘My sense of direction is very good’; perhaps self-evidently indicating that the direct question (e.g., ‘how well would you rate your sense of direction’) is the most effective way to solicit an ecological self-evaluation of an individual’s sense of direction.

The confirmatory factor analysis (see Appendix A, Table A6) generally depicts a similar pattern of item removal when items with the lowest factor loading are consecutively dropped until the 4-item version. Importantly, Items 1, 4, 14, and 15 demonstrate the strongest factor loadings and these factor loadings remain consistent from the 15-item version to the 4-item version. Lastly, the exploratory factor analysis with the final four items revealed that these items measured one factor based on eigenvalues being greater than 1. Factor loadings were strong for the four items; Item 1: 0.77, Item 4: 0.93, Item 14: 0.83, and Item 15: −0.81. Overall, this suggests that these four items measure one underlying factor and represent the strongest relationship with the construct of sense of direction.

In the R-SOD scale, a mean score of less than 11.5 is likely to be an individual with DTD whereas a score greater than or equal to 11.5 is likely to be a healthy control. For Item 1 (“I am very good at giving directions”), Item 4 (“My sense of direction is very good”), and Item 14 (“I can usually remember a new route after I have traveled it only once”), a score greater than 5.5 is likely to be an individual with DTD whereas a score less than 5.5 is likely to be a healthy control. For Item 15 (“I don’t have a very good “mental map” of my environment), a score less than 2.5 is likely to be an individual with DTD whereas a score greater than 2.5 is likely to be a healthy control. This model correctly identified 87% of DTD cases (sensitivity) whereas it correctly identified 71% of healthy individuals (specificity) with a balanced accuracy of 78%. Additionally, the area under the curve (AUC) is 79% which suggests acceptable discrimination between DTD and healthy individuals. Although, specificity is lower in this model, sensitivity is higher which suggests the ability to correctly identify DTD cases supporting the use of these items as a general screening tool for DTD. This trade-off between sensitivity and specificity is acceptable for the specific purpose of developing a tool to identify the presence of DTD. These scores will be implemented on our online platform (gettinglost.ca) to identify individuals that may potentially have DTD and will be translated into multiple languages.

## 4. Discussion

In this study, we identified a minimal number of four items from the Santa Barbara Sense of Direction (SBSOD) scale that would retain its sensitivity to DTD by distinguishing extremely well among DTD and healthy controls. We refer to this set of four items as the R-SOD scale. We provide evidence that the R-SOD scale exhibits excellent internal consistency (α = 0.900) and shows extremely similar patterns of sensitivity to sex differences in self-reported sense of direction, while remaining associated with a variety of objective computerized large-scale spatial orientation tasks that we have employed previously in assessing spatial abilities [16]. As such, we feel that the R-SOD (Appendix B) can effectively operate as a tool that is able to identify individuals affected by DTD, and given its brevity, could be of practical use in clinical and research settings where time is a constraint.

Our findings provide a descriptive, item-wise characterization of the original SBSOD scale items. Of these items, we identified a handful that their removal produced nominal improvements in scale wise performance. A 11-item version of the scale (see Figure 3) exhibited very high reliability (α = 0.932) with only nominal changes in group effects (DTD vs. controls) and associations with objective tasks as compared to the original scale. While the 4-item R-SOD largely retains these properties, the sole effective departure from the original scale properties is a reduction in association of the scale score with mental rotation performance, with the full SBSOD scale showing a *r_part_* = 0.154, dropping to *r_part_* = 0.098 with R-SOD scale. While we do not feel this undermines the utility of the shortened version of the scale, for research applications that are specifically interested in this association (which appears to be driven primarily by the ‘map-reading’ items 7 and 9 of the SBSOD scale) researchers may want to be cognizant of this change in scale properties as this scale may not be effective in identifying general spatial deficits. Conceptually, the association between map-reading and ecological navigation has presumably been decreasing with the high uptake of turn-by-turn GPS and other computerized navigational aids [38], which has decreased the need for more ‘traditional’ map-reading skills [39,40]. This increased reliance on GPS has been reported to have a negative effect on self-reported sense of direction [38,41] which may reduce the link between map reading and overall sense of direction. Nevertheless, the R-SOD and SBSOD scales share the same rank-ordered correlations with objective task performance.

Of some interest is the generally stronger associations between scale items and the SCT, as compared to those seen with the CMT; both tasks assess the large-scale spatial ability of forming a mental representation of the environment, with the CMT utilizing ecological stimuli as compared to the SCT utilizing abstract geometric ones. Superficially, a task with more ecologically relevant stimuli would be expected to generate a stronger association with a self-report scale that is ostensibly weighted towards real-life experiences. However, this was not the case in our study. The deviation may be partially driven by the better psychometric properties of the SCT as compared to CMT-like tasks, as previous work has shown that the SCT is more reliable [26], and the associations between self-report and CMT performance may be somewhat suppressed due to regression attenuation [42]. This possibility highlights a potential limitation of this work: while we are confident in our spatial battery, and have used it successfully in other research contexts, there is a wide variety of spatial assessments utilized by different researchers [43], and it is possible that using a different spatial assessment battery for validation would have produced evidence justifying a different pattern of selected items for a shortened scale. However, in the context of identifying DTD, which is known to rely mostly on the inability to form a cognitive map of the environment (which the SCT is measuring), the R-SOD scale remains a very valuable tool to be adopted. Additionally, despite the explicit request to report any neurological condition or cognitive difficulties aside from spatial orientation, since the current definition of DTD primarily relies on self-report measures, it remains a possibility that lifelong issues with orientation reported by some individuals may be experienced for other reasons. Another possible limitation in the DTD sample is that it is largely composed of females. This may be due to sampling bias where females are more likely to report difficulties or may reflect DTD being more prevalent in females. Lastly, we used a relatively straightforward method for cutting scale items. Although alpha is a commonly used measure to shorten scales, a more complex technique [44] or different selection criteria for the alpha [45] may have yielded different results. Nevertheless, the R-SOD retains excellent internal consistency and shows equivalent patterns of associations with our battery of spatial tasks that include assessments of small, medium, and large spatial abilities, supporting its utility as a rapid assessment of an individual’s self-reported sense of direction to potentially identify the presence of DTD. Future work is required to investigate the effectiveness of the R-SOD scale with a new group of participants to ensure reproducibility.

A recent DTD case study [18] highlighted healthcare providers focusing on navigation-related anxiety instead of the navigational difficulties experienced by the individual. This demonstrates a general lack of knowledge about DTD among healthcare providers which likely contributes to many potential cases of DTD being undetected for many years or misdiagnosed. It is crucial to increase awareness and knowledge about DTD, especially among healthcare providers and clinicians. The four items of the SBSOD scale that we identified in this study could easily be implemented in clinical settings for healthcare providers to utilize, and it may also be a useful tool for individuals who may suspect being affected by DTD.

## 5. Conclusions

In this study, we identified four items from the Santa Barbara Sense of Direction (SBSOD) scale which distinguish extremely well among DTD and healthy individuals. We have deemed this shortened scale as the Rapid Sense of Direction Scale (R-SOD). R-SOD can be of practical use as a tool to measure self-reported sense of direction to identify the presence of DTD in the general population.

## Figures and Tables

**Figure A1:**
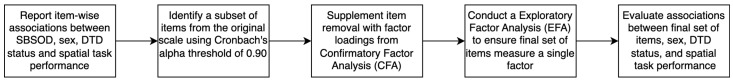
A flowchart depicting the statistical analysis procedure.

## Data Availability

The data and materials for all experiments are available at (DOI: 10.17605/OSF.IO/EY5NM, URL: osf.io/ey5nm/).

## Appendix A

**Table A1:** Reliability estimates (test–retest reliability and odd even reliability) for the spatial tasks MRT, 4MT, SCT, and CMT.

	Test–Retest Reliability	Odd-Even Reliability
Mental Rotation Task (MRT)		0.826
Four Mountains Task (4MT)		0.357
Spatial Configuration Task (SCT)	0.755	0.858
Cognitive Map Task (CMT)	0.517	

**Table A2:** Mean scores for each SBSOD item and the total overall scale score with standard deviation (*SD*). Positively stated items have their scores flipped so that a higher score corresponds to a better sense of direction.

	Healthy Male		Healthy Female		DTD Male		DTD Female	
Item	*M*	*SD*	*M*	*SD*	*M*	*SD*	*M*	*SD*
1	4.91	1.77	3.85	1.95	2.50	1.63	2.23	1.59
2	4.50	1.75	4.17	1.82	3.51	1.77	3.78	1.90
3	4.88	1.53	3.76	1.72	3.55	1.70	2.68	1.51
4	4.91	1.92	3.67	2.09	1.76	1.29	1.56	1.15
5	3.81	2.08	2.79	1.99	2.49	1.99	1.84	1.60
6	3.77	1.91	2.90	1.91	1.57	1.30	1.78	1.65
7	4.52	1.92	3.70	2.04	3.69	2.08	2.93	1.96
8	4.94	1.71	4.04	1.94	2.73	1.76	2.50	1.72
9	4.94	1.72	3.96	1.91	3.58	1.86	2.86	1.82
10	3.86	2.09	3.11	2.11	1.61	1.31	1.53	1.30
11	4.12	1.82	3.30	1.90	2.12	1.50	2.04	1.56
12	5.39	1.72	5.43	1.78	4.58	1.89	4.74	1.87
13	4.38	2.00	3.40	2.13	2.30	1.76	1.99	1.67
14	4.32	1.93	3.31	2.02	1.66	1.22	1.60	1.25
15	4.87	1.93	4.02	2.11	2.07	1.63	2.06	1.64
Overall Score	68.12	18.44	55.40	19.42	39.72	12.38	36.11	12.46

**Table A3:** Cohen’s *d* and *SE* values for sex (females and males) and group (DTD and healthy controls) for all 15 items of the scale and the overall SBSOD score. Positive Cohen’s *d* corresponds to males and healthy controls reporting a higher score (a better sense of direction) on each item.

	Sex		DTD	
Item	*d*	*SE*	*d*	*SE*
1	0.56	0.046	1.14	0.070
2	0.19	0.045	0.38	0.073
3	0.68	0.046	0.75	0.064
4	0.61	0.046	1.52	0.069
5	0.50	0.046	0.60	0.075
6	0.46	0.045	0.93	0.071
7	0.42	0.045	0.40	0.079
8	0.48	0.045	1.04	0.072
9	0.53	0.046	0.67	0.073
10	0.36	0.045	1.06	0.072
11	0.44	0.045	0.93	0.069
12	−0.03	0.045	0.42	0.072
13	0.48	0.045	0.91	0.077
14	0.51	0.046	1.28	0.068
15	0.41	0.045	1.26	0.075
Overall Score	0.67	0.046	1.44	0.660

**Table A4:** Partial correlations (controlled for sex and DTD) of each SBSOD item and the total scale score with nonspatial (Cambridge Face Memory Task (CFMT)) and spatial orientation tasks (Mental Rotation Task (MRT), Four Mountains Task (4MT), Spatial Configuration Task (SCT), and Cognitive Map Task (CMT)). Positive correlation coefficient indicates the tasks and items on the SBSOD move in the same direction, with a larger correlation coefficient suggesting a stronger relationship between tasks and items on the SBSOD.

Item	CFMT	MRT	4MT	SCT	CMT
1	0.04	0.08	0.11	0.15	0.09
2	0.06	0.03	0.08	0.07	0.02
3	0.06	0.03	0.03	0.05	−0.02
4	0.00	0.07	0.12	0.19	0.10
5	−0.02	0.09	0.09	0.08	0.02
6	−0.01	0.12	0.09	0.15	0.06
7	0.05	0.23	0.15	0.20	0.15
8	0.02	0.14	0.12	0.19	0.16
9	0.02	0.21	0.17	0.23	0.10
10	−0.03	0.00	0.03	0.08	0.00
11	0.02	0.10	0.11	0.17	0.11
12	0.05	0.00	0.05	0.03	0.07
13	0.02	0.15	0.13	0.13	0.07
14	0.00	0.09	0.12	0.17	0.09
15	0.05	0.08	0.13	0.20	0.13
Total Scale Score	0.04	0.15	0.17	0.23	0.13

**Table A5:** Properties of the shortened scales including Cohen’s D (sex), Cohen’s D (DTD), with nonspatial (Cambridge Face Memory Task (CFMT)) and spatial orientation tasks (Mental Rotation Task (MRT), Four Mountains Task (4MT), Spatial Configuration Task (SCT), Cognitive Map Task (CMT)), and internal consistency (Cronbach’s alpha). Positive Cohen’s *d* corresponds to males and healthy controls reporting a higher score (a better sense of direction) on each version. Positive correlation coefficient indicates the tasks and items on the SBSOD move in the same direction, with a larger correlation coefficient suggesting a stronger relationship between tasks and the overall SBSOD version. Item dropped indicates the item dropped in order to create the shortened scale in the corresponding row.

	Cohen’s D (Sex)	Cohen’s D (DTD)	CFMT	MRT	4MT	SCT	CMT	Alpha	Item Dropped
15 item	0.667	1.435	0.036	0.154	0.167	0.226	0.130	0.920	
14 item	0.669	1.435	0.030	0.156	0.163	0.225	0.134	0.925	2
13 item	0.695	1.433	0.026	0.161	0.162	0.229	0.131	0.931	12
12 item	0.675	1.444	0.030	0.159	0.160	0.232	0.136	0.931	5
11 item	0.666	1.493	0.025	0.137	0.149	0.220	0.122	0.932	7
10 item	0.632	1.494	0.018	0.142	0.155	0.228	0.132	0.931	3
9 item	0.617	1.527	0.017	0.123	0.144	0.214	0.127	0.929	9
8 item	0.613	1.532	0.020	0.118	0.144	0.214	0.131	0.926	6
7 item	0.604	1.549	0.019	0.103	0.137	0.215	0.132	0.921	13
6 item	0.598	1.571	0.017	0.097	0.133	0.209	0.124	0.915	11
5 item	0.617	1.569	0.029	0.114	0.151	0.226	0.152	0.907	10
4 item	0.608	1.576	0.030	0.098	0.145	0.215	0.131	0.900	8
3 item	0.573	1.568	0.021	0.094	0.144	0.217	0.131	0.889	1
2 item	0.604	1.535	0.002	0.091	0.132	0.201	0.109	0.868	15

**Table A6:** Factor loadings from a confirmatory factor analysis for Items 1–15. Item dropped indicates the item dropped in order to create the shortened scale in the corresponding row. A higher factor loading indicates a stronger relationship between the item and the underlying construct (sense of direction).

	Item Dropped	Item 1	Item 2	Item 3	Item 4	Item 5	Item 6	Item 7	Item 8	Item 9	Item 10	Item 11	Item 12	Item 13	Item 14	Item 15
**15 item**		0.808	−0.270	0.613	0.885	0.537	−0.675	0.504	−0.759	0.667	−0.715	−0.724	−0.290	−0.726	0.812	−0.803
**14 item**	2	0.808		0.613	0.886	0.538	−0.674	0.505	−0.759	0.667	−0.714	−0.724	−0.289	−0.727	0.812	−0.803
**13 item**	12	0.809		0.614	0.886	0.538	−0.674	0.504	−0.758	0.667	−0.713	−0.724		−0.725	0.812	−0.802
**12 item**	7	0.807		0.613	0.891	0.531	−0.675		−0.757	0.647	−0.720	−0.721		−0.718	0.818	−0.807
**11 item**	5	0.805		0.611	0.890		−0.676		−0.758	0.643	−0.725	−0.722		−0.716	0.819	−0.809
**10 item**	3	0.802			0.885		−0.682		−0.760	0.639	−0.728	−0.723		−0.718	0.820	−0.811
**9 item**	9	0.796			0.888		−0.683		−0.753		−0.737	−0.721		−0.708	0.825	−0.814
**8 item**	6	0.803			0.891				−0.748		−0.731	−0.722		−0.705	0.823	−0.815
**7 item**	13	0.800			0.894				−0.744		−0.733	−0.714			0.827	−0.815
**6 item**	11	0.779			0.906				−0.732		−0.732				0.837	−0.817
**5 item**	8	0.771			0.914						−0.725				0.842	−0.811
**4 item**	10	0.775			0.928										0.829	−0.801

## Appendix B. Rapid Sense of Direction (R-SOD) Scale

After reading each statement, please circle a number to indicate your level of agreement with the statement. Select “1” if you strongly disagree that the statement applies to you, “7” if you strongly agree with the statement, or some number in between if your agreement is intermediate. Select “4” if you neither agree nor disagree. When creating an overall score, the score for Item 4 must be reversed since it is a negatively worded statement.

1. I am very good at giving directions.

Strongly Disagree			Neither agree nor disagree			Strongly Agree
1	2	3	4	5	6	7

2. My “sense of direction” is very good.

Strongly Disagree			Neither agree nor disagree			Strongly Agree
1	2	3	4	5	6	7

3. I can usually remember a new route after I have traveled it only once.

Strongly Disagree			Neither agree nor disagree			Strongly Agree
1	2	3	4	5	6	7

4. I don’t have a very good “mental map” of my environment.

Strongly Disagree			Neither agree nor disagree			Strongly Agree
1	2	3	4	5	6	7
